# Effect of Parental–Child Age Gaps and Skipped-Generation Families on Comorbidities Related to Attention Deficit Hyperactivity Disorder: A Population-Based Case–Control Study

**DOI:** 10.3390/children12091123

**Published:** 2025-08-26

**Authors:** Hueng-Chuen Fan, Fang-Chuan Kuo, Jen-Yu Lee, Yu-Mei Chang, Kuo-Tung Chiang, Kuo-Liang Chiang

**Affiliations:** 1 Department of Pediatrics, Tungs Taichung Metroharbor Hospital, No. 699, Section 8, Taiwan Boulevard, Wuqi District, Taichung City 43503, Taiwan; fanhuengchuen@yahoo.com.tw; 2 Department of Life Sciences, Agricultural Biotechnology Center, National Chung Hsing University, Taichung City 402, Taiwan; 3 Department of Pediatrics, Tri-Service General Hospital, National Defense Medical Center, Taipei City 11243, Taiwan; 4 Department of Physical Therapy, Hungkuang University, No. 1018, Section 6, Taiwan Boulevard, Shalu District, Taichung City 433304, Taiwan; kfc@hk.edu.tw; 5 Department of Statistics, Feng Chia University, No. 100, Wenhua Rd., Xitun District, Taichung City 407102, Taiwan; jylee@fcu.edu.tw; 6 Department of Statistics, Tunghai University, No. 1727, Section 4, Taiwan Boulevard, Xitun District, Taichung City 407224, Taiwan; yumei0115@thu.edu.tw; 7 Department of Psychiatry, Beitou Branch, Tri-Service General Hospital, National Defense Medical Center, No. 60, Xinmin Rd., Beitou District, Taipei City 11243, Taiwan; 809010016@mail.ndmctsgh.edu.tw; 8 Giraduate Institution of Medical Science, National Defense Medical Center, No. 161, Section 6, Minquan E. Rd., Nehu District, Taipei City 11490, Taiwan; 9 Department of Pediatric Neurology, Kuang-Tien General Hospital, No. 117, Shatian Road, Shalu District, Taichung City 433, Taiwan; 10 Department of Nutrition, Hungkuang University, No. 1018, Section 6, Taiwan Boulevard, Shalu District, Taichung City 433304, Taiwan

**Keywords:** attention deficit hyperactivity disorder (ADHD), skipped-generation families, comorbidities, maternal age at childbirth (MACB), paternal age at childbirth (PACB)

## Abstract

**Highlights:**

**What are the main findings?**

- Older parental age is linked to higher risks of ASD and bipolar disorder in children with ADHD.
- Skipped-generation care increases emotional and behavioral comorbidities.

**What are the implications of the main findings?**

- Parental age and caregiving structure are key risk factors for ADHD comorbidities.
- Early screening may help guide targeted interventions.

**Abstract:**

**Background**: While attention deficit/hyperactivity disorder (ADHD) is characterized by neurodevelopmental heterogeneity, the influence of familial structural factors—particularly parental age and skipped-generation caregiving—on comorbidity patterns remains insufficiently studied. This study examined the associations between parent–child age gaps, skipped-generation family structures, and psychiatric comorbidities in children with ADHD. **Methods**: Data came from Taiwan’s NHIRD (2009–2013), including 79,163 ADHD cases and 395,815 matched controls. Key variables included maternal and paternal age at childbirth and grandparent-paid insurance premiums as a proxy for skipped-generation caregiving. Conditional logistic regression was used to estimate odds ratios (ORs) for 20 psychiatric and developmental comorbidities. **Results**: Children with ADHD exhibited significantly higher odds of various comorbidities, including oppositional defiant disorder (OR = 147.05, 95% CI = 101.0–214.1), somatoform disorder (OR = 25.78, 95% CI = 7.96–83.46), anxiety disorder (OR = 24.49, 95% CI = 17.9–33.5), emotional disturbances during childhood and adolescence (OR = 13.99, 95% CI = 9.15–21.4), and autism spectrum disorder (OR = 8.07, 95% CI = 6.63–9.82). Advanced maternal age (>35 years) was associated with increased odds of autism spectrum disorder (OR = 1.47, 95% CI: 1.29–1.67) and speech/language delay (OR = 1.33, 95% CI: 1.17–1.52), whereas younger maternal age (≤25 years) was linked to higher odds of anxiety disorder (OR = 1.23, 95% CI: 1.13–1.33) and adjustment reaction (OR = 1.41, 95% CI: 0.95–2.11). Maternal age under 20 years showed the highest odds for bipolar disorder (OR = 2.01, 95% CI: 1.04–3.88). For paternal age, older age (>35 years) was associated with increased odds of autism (OR = 1.14, 95% CI: 1.04–1.26) and speech/language delay (OR = 1.15, 95% CI: 1.04–1.27), whereas paternal age ≤20 years was strongly linked to bipolar disorder (OR = 3.58, 95% CI: 1.54–8.32) and anxiety (OR = 1.39, 95% CI: 1.01–1.93). Children from skipped-generation families—defined as grandparent-paid insurance premiums without parental cohabitation—had significantly higher odds of bipolar disorder (OR = 2.88, 95% CI: 1.36–6.11), personality disorder (OR = 9.23, 95% CI: 2.23–38.20), adjustment reaction (OR = 2.23, 95% CI: 1.39–3.59), and emotional disturbances during childhood/adolescence (OR = 1.69, 95% CI: 1.13–2.54). **Conclusions**: Both extremes of parental age and skipped-generation caregiving are linked to specific associations with certain psychiatric comorbidity patterns in children with ADHD. These findings highlight the importance of integrating family structure into diagnostic assessments and treatment planning and support the development of targeted early interventions.

## 1. Introduction

Attention deficit/hyperactivity disorder (ADHD) represents one of the most prevalent neurodevelopmental disorders in pediatric populations, affecting approximately 8.0% (95% CI: 6.0–10%) of children and adolescents globally [1]. The disorder is characterized by persistent and impairing patterns of inattention, hyperactivity, and impulsivity that significantly impact academic, social, and family functioning. While diagnostic prevalence varies according to applied criteria and population characteristics, the substantial burden of ADHD extends well beyond childhood, with longitudinal studies demonstrating symptom persistence into adulthood in a significant proportion of cases [2].

The clinical presentation of ADHD is marked by considerable heterogeneity, with affected children frequently presenting with complex comorbidity profiles encompassing psychiatric, developmental, and cognitive conditions. Common comorbidities include autism spectrum disorder (ASD), intellectual disability, anxiety disorders, oppositional defiant disorder, speech and language impairments, and mood disorders [3,4]. This comorbidity complexity substantially complicates diagnostic processes and treatment planning, necessitating comprehensive, individualized assessment approaches that extend beyond the core ADHD symptomatology.

Genetic contributions to ADHD are well established, with genome-wide association studies, rare variant analyses, and twin studies supporting a strong heritable component across diverse populations [5,6,7,8,9]. These findings highlight the role of both common and rare risk alleles, polygenic burden, and gene–environment interactions in shaping ADHD phenotypes. Neurobiological studies implicate alterations in fronto-striatal circuitry, dopaminergic and noradrenergic dysregulation, and atypical neural connectivity [10,11,12,13,14,15,16,17,18], while environmental factors—including prenatal exposures, early-life adversity, and socioeconomic disadvantage—have also been linked to ADHD onset and persistence [19,20,21,22,23,24,25,26,27]. Collectively, genetic predisposition and environmental exposures jointly influence ADHD’s heterogeneous presentation and comorbidity patterns.

However, familial structural variables—particularly parental age at conception and alternative caregiving arrangements—remain inadequately characterized despite their potential significance for comorbidity risk and treatment outcomes. Advanced parental age has been associated with increased risk for various neurodevelopmental conditions, including ASD and schizophrenia, potentially mediated through de novo mutations, epigenetic modifications, or psychosocial stressors [28,29,30,31,32]. Conversely, younger parental age correlates with socioeconomic disadvantage, reduced parenting preparedness, and elevated risk for childhood emotional and behavioral difficulties [33,34,35,36,37].

Of particular relevance to pediatric populations in rapidly developing economies is the emergence of skipped-generation family structures, wherein grandparents assume primary caregiving responsibilities due to parental absence related to employment demands or family dissolution [38]. These arrangements present unique developmental challenges, as generational disparities in child-rearing philosophies, caregiver stress, and reduced parental involvement may contribute to emotional and behavioral dysregulation—particularly problematic for children with ADHD, who demonstrate heightened sensitivity to environmental stressors [39].

Despite growing recognition of grandparental caregiving arrangements, limited research has examined their specific impact on neurodevelopmental outcomes in children with ADHD. Factors including generational differences in behavioral management approaches, caregiver mental health status, and unfamiliarity with contemporary evidence-based interventions may significantly influence developmental trajectories in this vulnerable population.

Potential Mechanistic Pathways Linking Familial Structure to ADHD Comorbidities: Advanced parental age may predispose children to neurodevelopmental conditions such as ASD, language delays, and cognitive impairments through biological pathways including de novo mutations, epigenetic alterations, and age-related gamete changes. In contrast, younger parental age and skipped-generation caregiving may influence emotional and behavioral outcomes via psychosocial mechanisms such as caregiver stress, limited parenting preparedness, and generational gaps in child-rearing practices. These biological and psychosocial factors likely interact with a child’s genetic vulnerability, contributing to the heterogeneity of ADHD comorbidity profiles.

The current epidemiological literature on ADHD comorbidities has predominantly focused on clinical phenotyping, neurocognitive characterization, and pharmacological response patterns. Population-based investigations examining how parental demographic factors and alternative family structures influence ADHD comorbidity profiles remain scarce. An enhanced understanding of these contextual familial determinants is essential in developing targeted early intervention strategies and personalized treatment approaches.

Hypothesis: Grounded in the existing literature and a bio-psychosocial framework, we hypothesized that advanced parental age would increase the risk of autism spectrum disorder (ASD) and language delays via biological mechanisms, whereas younger parental age and skipped-generation care would be associated with emotional or behavioral comorbidities due to psychosocial stressors or generational gaps in parenting.

Study objectives: Using Taiwan’s National Health Insurance Research Database (NHIRD), this large-scale, population-based case–control study aimed to achieve the following:

1. Examine associations between maternal and paternal age at childbirth and specific psychiatric and developmental comorbidities in children and adolescents with ADHD;
2. Investigate the impact of skipped-generation family structures—conceptually defined as households with grandparent-paid insurance premiums and without parental cohabitation—on ADHD comorbidity patterns;
3. Identify distinct comorbidity profiles associated with parental age extremes and grandparental caregiving to inform clinical risk stratification and family-centered intervention approaches.

By integrating familial structural factors into ADHD comorbidity analysis, this investigation seeks to advance a more comprehensive framework for understanding and managing this complex neurodevelopmental disorder in pediatric populations.

## 2. Materials and Methods

### 2.1. Data Source

This study utilized the NHIRD, which includes medical records for nearly 99% of Taiwan’s population. We accessed outpatient and inpatient records from 2009 to 2013. ADHD diagnoses were based on International Classification of Diseases, Ninth Revision, Clinical Modification (ICD-9-CM) code 314 and aligned with Diagnostic and Statistical Manual of Mental Disorders, Fourth Edition (DSM-IV) criteria. To enhance diagnostic accuracy, we included cases with records from medical centers, psychiatric hospitalizations, or catastrophic illness registrations.

### 2.2. Study Population

We identified 79,163 children under 18 years with at least one ADHD-related medical claim. Each case was matched with 5 controls by age (±30 days) and sex, yielding 395,815 controls. Parental age at childbirth was derived from linked birth records. Skipped-generation caregiving was identified when a child’s insurance premium was paid by grandparents and the child was not registered in the same household as the parents.

### 2.3. Comorbidity Definitions

ADHD-related comorbidities were identified using ICD-9-CM codes, which were reviewed and validated by two pediatric neurologists and one psychiatrist. We examined 20 common psychiatric and developmental conditions, including ASD, speech/language disorders, intellectual disability, mood disorders, learning disabilities, and sleep disturbances; the corresponding diagnostic codes are listed in Table S1.

Although the use of claims-based codes may raise concerns about diagnostic validity, prior NHIRD validation studies have demonstrated acceptable accuracy for psychiatric diagnoses. A structured chart review study reported high positive predictive values (≥0.70) and excellent interrater reliability for psychotic and affective disorders, supporting the general reliability of NHIRD psychiatric codes [40]. Furthermore, an ADHD-specific NHIRD investigation noted that most ADHD diagnoses are made by board-certified psychiatrists, which enhances reliability [41].

### 2.4. Definition of Parent–Child Age Gap and Skipped-Generation Family

The parent–child age gap was determined based on maternal and paternal ages at the time of childbirth. This information was obtained indirectly by linking the personal identification numbers of the enrolled individuals and controls to their parental records. Maternal age at childbirth (MACB) and paternal age at childbirth (PACB) were each categorized into five stratified groups: ≤20, 21–25, 26–30, 31–35, and >35 years. A skipped-generation family was conceptually defined as one with grandparent-paid insurance premiums and no parental cohabitation. Operationally, it was defined as a household in which the child’s National Health Insurance premiums were paid by grandparents, with no parental cohabitation recorded in the official household registry. Cases with missing parental age or insurance data were retained in the main analysis but excluded from subsequent stratified analyses involving these variables. This study followed the STROBE guidelines for observational research. The completed checklist is provided in the Supplement Table S8.

### 2.5. Statistical Analysis

Descriptive statistics and chi-squared tests were used to compare demographic variables. Conditional logistic regression was applied to estimate ORs and 95% confidence intervals (CIs) for comorbidities associated with parental age and skipped-generation caregiving. *t*-tests assessed mean parental age differences for each comorbidity. Analyses were performed using R (version 3.6.2).

## 3. Results

### 3.1. Demographic Characteristics

Table 1 presents the characteristics of the ADHD case and control groups. Among the 79,163 patients with ADHD, 79.42% were male, and 57.51% were aged 7–12 years. The proportions of maternal age at childbirth (MACB), paternal age at childbirth (PACB), and skipped-generation families were significantly higher in the case group compared to the control group (*p* < 0.01).

### 3.2. ADHD Comorbidities

**Figure 1:**
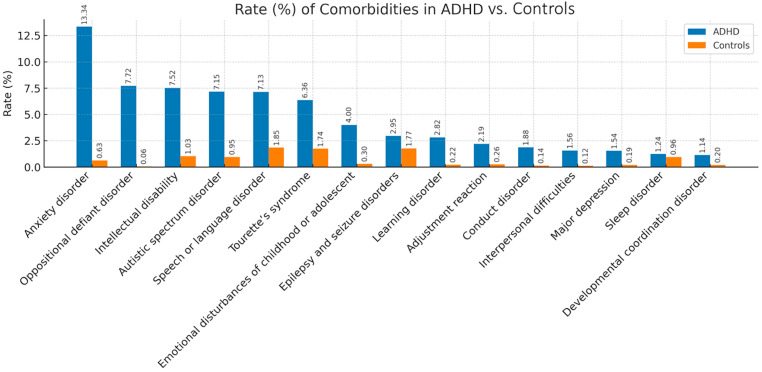
Rates of the most common comorbidities among children and adolescents with ADHD compared to those in the control group in Taiwan from 2009 to 2013.

Figure 1 illustrates the rates of the most common comorbidities in the ADHD case group compared to the control group in Taiwan from 2009 to 2013. These comorbidities include anxiety disorders, oppositional defiant disorder (ODD), intellectual disability, autism spectrum disorder (ASD), speech or language disorders, and Tourette syndrome. The rates of the 15 most common comorbidities were significantly higher in the ADHD group than in the control group (*p* < 0.05). Detailed numbers and proportions of all comorbidities across different age groups in children with ADHD are provided in Table S2.

**Figure 2:**
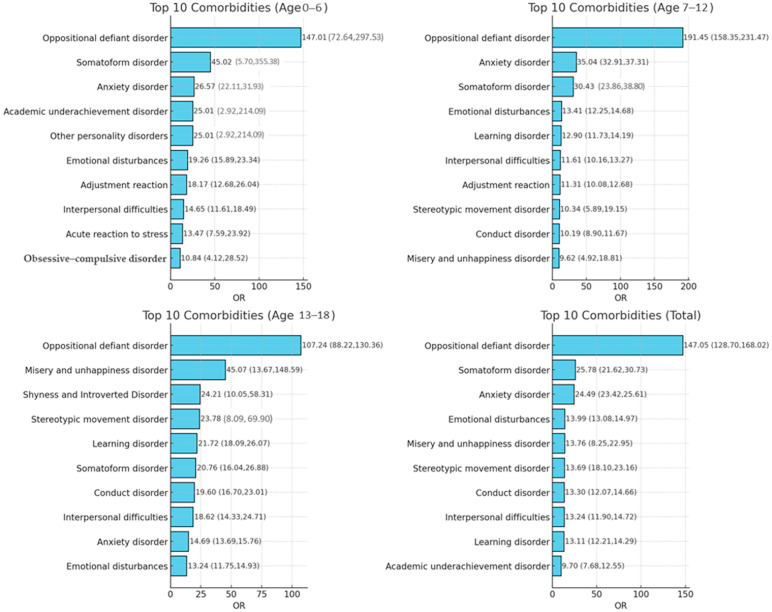
Top 10 comorbidities in patients with ADHD, overall and by age group. Odds ratios (ORs) with 95% CIs are presented for each subgroup (for the complete version, please see Supplementary Table S3).

We present the results of the conditional logistic regression analysis used to calculate odds ratios (ORs) for various comorbidities (see Table S3 for detailed results). Figure 2 summarizes the ten most strongly associated comorbidities in the overall ADHD cohort and in each age-stratified subgroup. Across the entire ADHD cohort, the five comorbidities with the highest ORs were oppositional defiant disorder (ODD), somatoform disorder, anxiety disorder, emotional disturbances, and misery and unhappiness disorder (Figure 2). The pattern of strongly associated comorbidities varied by age group:

- Age 0–6 years: ODD, somatoform disorder, anxiety disorder, academic underachievement disorder, and other personality disorders;
- Age 7–12 years: ODD, anxiety disorder, somatoform disorder, emotional disturbances, and learning disorder;
- Age 13–18 years: ODD, misery and unhappiness disorder, shyness and introverted disorder, stereotypic movement disorder, and learning disorder.

Notably, several conditions—such as sexual deviations and disorders, eating problems, schizophrenia, drug addiction, and substance abuse—demonstrated higher ORs in adolescents (13–18 years) compared with younger groups.

**Figure 3:**
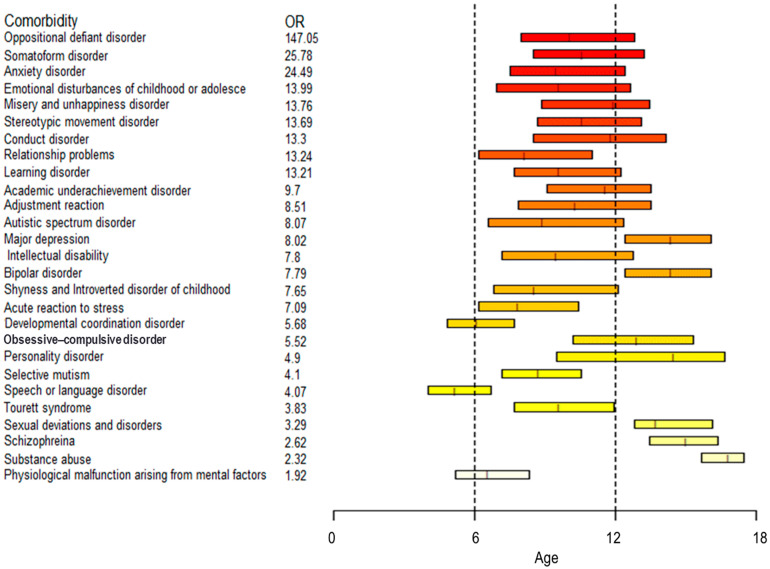
A timeline of common comorbidities (OR > 1.5, number > 10) according to the median, quartile 1, and quartile 3 of the age of the patients with ADHD at the time when patients with ADHD received the first diagnosis of these comorbidities. The color gradient corresponds to the odds ratio (OR), with red indicating higher ORs and lighter shades indicating lower ORs.

Figure 3 depicts a timeline of common comorbidities with OR > 1.5 and case counts >10, based on the median, first quartile (Q1), and third quartile (Q3) ages at first diagnosis. Speech or language disorders appeared earliest, while substance abuse was typically diagnosed last.

### 3.3. Impact of Parent–Child Age Gaps and Skipped-Generation Families on Common Comorbidities in Children with ADHD

**Figure 4:**
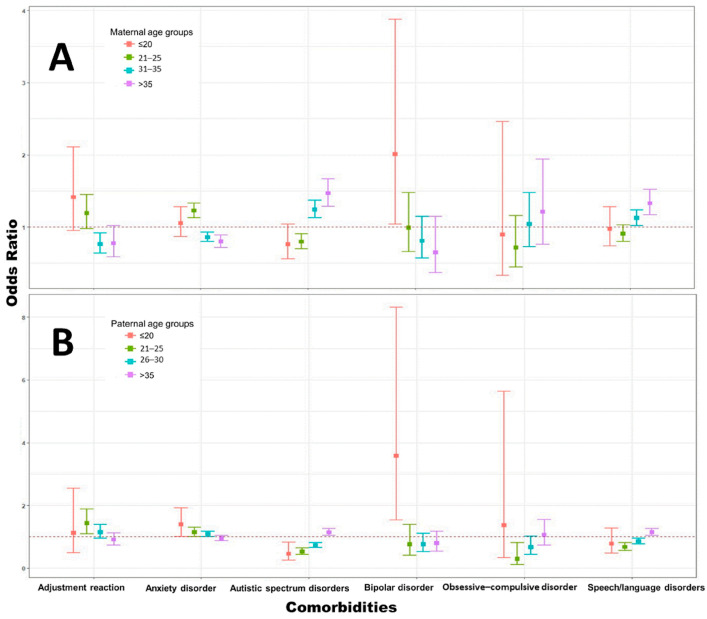
(**A**) The OR results of the six statistically significant comorbidities stratified by maternal age at childbirth (MACB), referenced by age 26–30 group. (**B**) The OR results of the six statistically significant comorbidities stratified by paternal age at childbirth (PACB), referenced by age 31–35 group.

Anxiety, adjustment reaction, obsessive–compulsive disorder (OCD), autism spectrum disorder (ASD), speech or language delay, and bipolar disorder were the six comorbidities that showed significant associations with the mean maternal or paternal age at childbirth (MACB or PACB) in the *t*-test analysis. The corresponding odds ratio (OR) results are presented in Figure 4A,B.

In the MACB analysis (reference group: 26–30 years), the ORs for anxiety and adjustment reaction showed a negative trend with increasing maternal age, whereas the ORs for ASD, OCD, and speech or language delay demonstrated positive trends. Among these, only anxiety, ASD, and speech or language delay reached statistical significance in the stratified analysis.

Figure 4B presents the PACB analysis (reference group: 31–35 years), which showed similar OR trends to the MACB analysis. Likewise, only anxiety, ASD, and speech or language delay remained statistically significant in the stratified analysis.

Notably, bipolar disorder was significantly more prevalent in the <20-year parental age group compared to other age groups, regardless of whether the data were stratified by MACB or PACB. Detailed statistical results are provided in Tables S4–S7.

Table 2 summarizes the chi-squared test and OR results for common ADHD comorbidities in relation to skipped-generation families. Four comorbidities—personality disorder, bipolar disorder, adjustment reaction, and emotional disturbances of childhood or adolescence—were significantly more prevalent in the ADHD group than in the control group.

**Figure 5:**
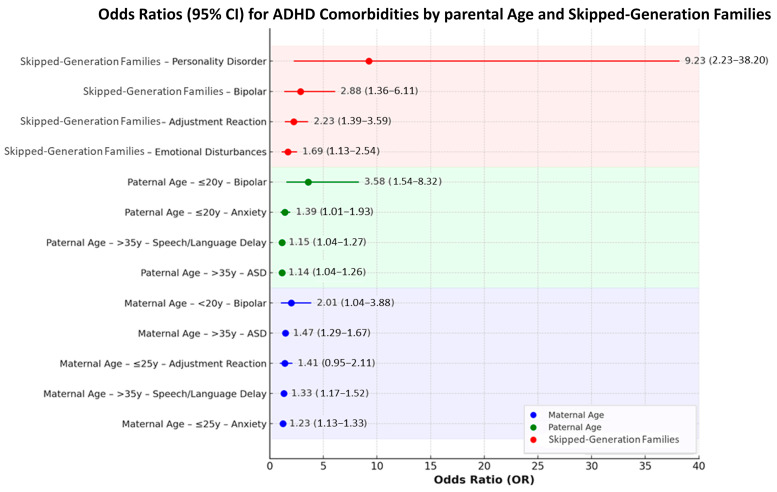
Summary of odds ratios (ORs) and 95% confidence intervals (CIs) for significant ADHD comorbidities associated with maternal age, paternal age, and skipped-generation status. The three panels are color-coded by factor: blue for maternal age, green for paternal age, and red for skipped-generation status. Within each panel, comorbidities are ranked from highest to lowest OR for visual clarity.

Summary of Key Associations: Figure 5 summarizes the odds ratios for statistically significant comorbidities associated with maternal age, paternal age, and skipped-generation status, ranked by effect size for ease of comparison. Advanced maternal age (>35 years) was associated with higher odds of autism spectrum disorder (OR = 1.47, 95% CI: 1.29–1.67) and speech/language delay (OR = 1.33, 95% CI: 1.17–1.52), whereas younger maternal age (≤25 years) was linked to anxiety disorder (OR = 1.23, 95% CI: 1.13–1.33) and adjustment reaction (OR = 1.41, 95% CI: 0.95–2.11). Maternal age under 20 years showed the highest odds for bipolar disorder (OR = 2.01, 95% CI: 1.04–3.88). 

For paternal age, older age (>35 years) was related to autism (OR = 1.14, 95% CI: 1.04–1.26) and speech/language delay (OR = 1.15, 95% CI: 1.04–1.27), whereas paternal age ≤20 years was strongly associated with bipolar disorder (OR = 3.58, 95% CI: 1.54–8.32) and anxiety disorder (OR = 1.39, 95% CI: 1.01–1.93).

Children from skipped-generation families—defined as grandparent-paid insurance premiums without parental cohabitation—had notably higher odds of personality disorder (OR = 9.23, 95% CI: 2.23–38.20), bipolar disorder (OR = 2.88, 95% CI: 1.36–6.11), adjustment reaction (OR = 2.23, 95% CI: 1.39–3.59), and emotional disturbances during childhood/adolescence (OR = 1.69, 95% CI: 1.13–2.54).

## 4. Discussion

In epidemiological studies, ADHD is most commonly diagnosed in children and adolescents, with peak diagnosis typically occurring between ages 6 and 12, followed by a decline in prevalence. Males are disproportionately affected, with reported male-to-female ratios ranging from 2:1 to 4:1 [3,42], consistent with our findings. In our cohort, the annual ADHD prevalence ranged from 2.27% to 5.11% between 2009 and 2013—a rate markedly lower than global estimates [1].

We hypothesize that many children with ADHD during this period may not have received appropriate clinical evaluation, diagnosis, or treatment. As the National Health Insurance Research Database (NHIRD) only captured retrospective data on individuals who sought medical care, undiagnosed cases may have been missed. Limited awareness of ADHD among parents, teachers, or guardians could result in misattribution of symptoms to behavioral issues, thereby missing the optimal window for intervention and contributing to underdiagnosis.

Beyond its prevalence, ADHD significantly affects multiple domains of daily functioning, including academic performance, occupational success, interpersonal relationships, and overall mental health [43,44]. It is frequently associated with various comorbidities. Our analysis revealed significantly elevated ORs for psychiatric, neurological, and developmental–cognitive disorders; learning difficulties; sleep disorders; and interpersonal problems. The age of onset, prevalence, and ORs of these comorbidities are consistent with previous reports [45,46,47,48,49]. Notably, we observed an increased prevalence of sexual behavior problems, anorexia nervosa, schizophrenia, drug addiction, and substance abuse among adolescents with ADHD. Some cases diagnosed with bipolar disorder may have represented disruptive mood dysregulation disorder (DMDD), a condition added to the DSM in 2013, which shares overlapping symptoms with bipolar disorder in adolescence but differs in etiology, treatment, and prognosis [50,51].

In contrast to prior studies [52,53,54,55], we found weaker associations between ADHD and adverse outcomes such as trauma, burn injuries, suicide, and traffic accidents. This discrepancy may reflect the limited follow-up period of our study, as such events often occur later in adolescence or adulthood. Additionally, many ADHD-diagnosed adolescents in the NHIRD cohort were receiving pharmacological treatment, potentially mitigating adverse outcomes. Misclassification may also have occurred if control subjects had undiagnosed or untreated ADHD. Finally, the use of administrative data may limit the accuracy of comorbidity detection. Nevertheless, other NHIRD-based studies with longer follow-up and medication data have reported such associations [52,56].

Our estimates for common comorbidities of ADHD have certain limitations. First, some strata contained zero events, precluding the computation of valid CIs using standard logistic regression. In these cases, we applied exact methods or labeled the result as “not estimable”, an issue more common for rare comorbidities in certain age groups. Second, some CIs were wide, indicating substantial imprecision. For example, in the 0–6-year-old group, somatoform disorder occurred in 9 of 16,869 ADHD cases and 1 of 84,345 controls, yielding an OR of 45.02 (95% CI 5.70–355.38; Fisher’s exact test *p* = 8.42 × 10^−7^). Although statistically significant, the wide CI reflects the very small number of events, warranting cautious interpretation (Table S3). Third, we did not adjust for multiple comparisons. Examining 20 comorbidities without correction increases the likelihood of type I error. Our primary aim, however, was to describe the overall comorbidity profile of ADHD rather than to test specific hypotheses for each comorbidity. This choice nonetheless warrants cautious interpretation of nominal *p*-values, particularly for associations with borderline statistical significance.

Regarding parental age, a meta-analysis reported a U-shaped association between maternal or paternal age and ADHD risk, with younger parental age showing increased risk (adjusted ORs: 1.49 for maternal, 1.75 for paternal age), while advanced age showed no significant association [57]. Interestingly, both maternal and paternal ages were significantly higher in the ADHD group compared to controls in our cohort (Table 1). A Danish registry study similarly reported increased hyperkinetic disorder risk with both younger and older paternal age [30].

Several studies have investigated the relationship between parental age and neurodevelopmental outcomes. In our data, both maternal and paternal age at childbirth (MACB and PACB) were associated with higher ORs for autism spectrum disorder (ASD) and speech impairment, aligning with findings from a single-institution study in Northern California [58]. A meta-analysis of 16 studies found an adjusted relative risk of 1.52 for autism among children of mothers aged >35 compared to those aged 25–29 [59]. The Western Australian Pregnancy Cohort Study also found advanced maternal age was associated with increased risk of depression, anxiety, and stress in female offspring by age 20 [60]. However, some evidence suggests older maternal age may confer protective effects on certain cognitive and behavioral outcomes [61]. Advanced paternal age has been linked to increased risks of psychiatric disorders, particularly ASD and schizophrenia [30,62,63,64].

Our finding that younger maternal age is associated with anxiety and adaptation problems in offspring aligns with the literature suggesting that children born to younger mothers face higher risks of cognitive delays, socioemotional challenges, behavioral problems, academic underperformance, and increased smoking and alcohol use—even after adjusting for socioeconomic variables [65,66]. The Danish registry study further reported stronger associations between younger maternal age and anxiety/stress-related disorders than with paternal age [30]. Additionally, children born to parents under 20 exhibited higher ORs for bipolar disorder, although this finding contrasts with most of the literature, which more often links bipolar disorder to advanced parental age [61,62]. Only one prior study has reported a similar association with younger parental age [30].

While most ADHD research has focused on parental demographic characteristics, relatively few studies have addressed the potential impact of grandparental caregiving. In contemporary Taiwanese society, evolving socioeconomic conditions—such as increased female labor force participation, rising divorce rates, and greater geographic mobility—have contributed to a growing reliance on grandparents for childcare. In such caregiving arrangements, generational gaps and shifting social norms may influence parenting styles and, in turn, increase the risk of psychological and behavioral challenges in children. These changes are not unique to Taiwan. Across industrialized nations, household structures have undergone significant transformations over the past several decades. Key demographic shifts—including below-replacement fertility, rising divorce rates, population aging, and international migration—have contributed to a decline in the traditional nuclear family model. The global proportion of nuclear households decreased from 63.6% in 1990 to 54.3% in 2010, while the prevalence of single-parent and skipped-generation households increased from 5.8% and 0.9% to 7.5% and 1.4%, respectively [67]. These alternative family structures have become increasingly common and may exert differing influences on children’s development. In Taiwan, the number of skipped-generation households rose from approximately 75,000 in 2001 to 114,000 in 2018, based on statistics from the Gender Equality Committee’s Executive, Yuan [38,68]. Similarly, the number of single-parent households increased from 520,000 to over 830,000 during the same period. These trends underscore the growing importance of understanding the developmental implications of non-traditional family structures in both global and regional contexts.

Evidence from developmental psychology and neuroscience suggests that the family environment plays a foundational role in shaping children’s cognitive, emotional, and behavioral outcomes. In nuclear families, parents are typically the primary caregivers and are more likely to provide consistent and developmentally appropriate support. Studies have shown that parental characteristics—such as effort, perseverance, emotional responsiveness, and verbal engagement—can positively influence children’s cognitive skills, emotional regulation, and academic performance [69,70,71,72]. By contrast, children raised in non-traditional caregiving contexts—such as by grandparents—may experience different developmental trajectories. Grandparent caregivers, while often providing essential support, may be limited by lower educational levels, outdated parenting practices, or financial constraints, all of which could impact caregiving quality. Although some studies suggest that grandparental involvement can mitigate stress in single-parent or stepfamily households [73,74], others indicate that it may be associated with increased emotional or behavioral problems, particularly when compounded by grandparental mental health issues [75].

Although most comparative studies focus on nuclear versus a single non-traditional family structure (e.g., single-parent households), there remains no clear consensus on which caregiving arrangement poses the greatest risk for adverse developmental outcomes. Clarifying these associations is essential not only for advancing scientific understanding but also for guiding the development of targeted, evidence-based policy interventions aimed at supporting children in diverse family environments.

Although ODD exhibits a high comorbidity rate among children with ADHD, our findings did not demonstrate a significant association between ODD and either parental age or skipped-generation caregiving. This suggests that the emergence of ODD may be more strongly driven by intrinsic ADHD-related behavioral dysregulation and shared neurobiological mechanisms rather than by family structure alone [76,77].

To our knowledge, this is the first comprehensive study to examine the association between maternal/paternal age at childbirth and grandparental caregiving (skipped-generation families) with ADHD comorbidities. Our findings indicate that grandparental caregiving is modestly associated with a higher prevalence of certain comorbidities—specifically, personality disorders, bipolar disorder, adjustment problems, and emotional disturbances—but not with most other comorbidities. This may reflect caregiver stress, limited parenting preparedness, and generational gaps in child-rearing. Supporting evidence indicates that grandparents as primary caregivers are more prone to depression [78], which—particularly after parental divorce—can increase grandchildren’s risk of anxiety [79]. These results suggest that the mental health and caregiving style of grandparents may shape emotional and behavioral outcomes in grandchildren. Nevertheless, other studies suggest grandparental involvement can sometimes buffer against such problems in children in single-parent or stepfamily contexts [73,80].

A key strength of our study is the use of a large, nationwide claims database, enabling indirect assessment of caregiving-related risk factors for psychoemotional comorbidities in ADHD. A primary limitation is the reliance on ICD-9-CM diagnostic codes without direct validation against clinical records, which may introduce potential misclassification of ADHD and comorbidities. While a chart review was not feasible, given the large-scale design, previous validation work within the Taiwan NHIRD provides important context: Wang et al. [40] reported high positive predictive values (≥0.70) and excellent interrater reliability for psychiatric diagnoses, and Liu et al. [41] noted that most ADHD diagnoses are made by board-certified psychiatrists. These findings support the overall validity of our case definitions, though residual misclassification remains possible.

Additional limitations include the following: First, ADHD subtypes could not be distinguished, though this is unlikely to affect demographic associations. Second, as a secondary data source, the NHIRD is subject to limitations in diagnostic accuracy and completeness. Third, our study did not account for urban–rural differences in family structures, which may significantly influence our findings. Rural grandparents often face compounded socioeconomic constraints, including limited healthcare access and lower educational attainment, that may differentially impact ADHD comorbidity profiles compared to urban settings where migrant parental labor creates different caregiving arrangements [81,82]. Fourth, certain key contextual variables—including SES (e.g., proxy-insured premium levels), environmental exposures, and medication usage history—were not included in our analysis. Although the NHIRD contains certain proxy SES indicators and other relevant data, these were not requested in our original application and were therefore unavailable for the present study. Fifth, skipped-generation families may sometimes experience medical care challenges, as grandchildren cared for by grandparents might be neglected regarding physical health problems [83]. This is particularly relevant because several physical conditions can mimic ADHD symptoms, potentially leading to diagnostic confusion. Future research should consider incorporating these variables and applying propensity score matching for SES to help minimize residual confounding, as well as incorporating geographic stratification and directly assessing grandparental mental health, parenting style, and household SES to validate the mechanisms underlying skipped-generation-related emotional disturbances.

## 5. Conclusions

Extremes of parental age and skipped-generation caregiving are significantly associated with distinct comorbidity profiles in children with ADHD. While familial factors may inform clinical risk assessment, the observed associations do not establish causality, and unmeasured confounding factors may exist. Further research is warranted to clarify underlying mechanisms and to confirm these findings in diverse populations. In the interim, clinicians may consider targeted screening—for example, assessing bipolar disorder risk in children with ADHD with very young parents or skipped-generation caregiving—as part of comprehensive care planning.

Extremes of parental age and skipped-generation caregiving are associated with certain comorbidity patterns in children with ADHD. While familial factors may contribute to risk assessment considerations, these associations should be interpreted cautiously due to the observational nature of the study and potential unmeasured confounding. Further research is warranted to clarify the underlying mechanisms and to confirm these findings in diverse populations. In the interim, clinicians might consider targeted screening—for example, assessing bipolar disorder risk in children with ADHD with very young parents or skipped-generation caregiving—as part of comprehensive care planning, while recognizing that current evidence does not yet justify the broad implementation of personalized management strategies.

## Figures and Tables

**Table 1:** Demographic characteristics of patients with ADHD and controls.

	ADHD Patients		Control Group		*p*-Value
	n = 79,163	Percentile	n = 395,815	Percentile	
Characteristic					
Sex					
Female	16,295	20.58%	81,475	20.58%	
Male	62,868	79.42%	314,340	79.42%	1.00
Age (years)					
0–6	16,869	21.31%	84,345	21.31%	
7–12	45,524	57.51%	22,7620	57.51%	
13–18	16,770	21.18%	83,850	21.18%	1.00
* Maternal age					
≤20	900	2.47%	5329	2.85%	
21–25	6175	16.92%	34,588	18.5%	
26–30	14,538	39.83%	76,240	40.78%	
31–35	11,057	30.29%	54,411	29.1%	
>35	3834	10.5%	16,403	8.77%	<0.01
* Paternal age					
≤20	270	0.85%	1669	1.04%	
21–25	2516	7.92%	14,311	8.93%	
26–30	9327	29.38%	47,636	29.72%	
31–35	11,266	35.48%	56,313	35.13%	
>35	8371	26.37%	40,364	25.18%	<0.01
* Skipped-generation family					
Yes	381	0.48%	1637	0.41%	
No	78,782	99.52%	394,178	99.59%	<0.01

* Maternal age, maternal age at child birth; paternal age, paternal age at child birth; skipped-generation family, grandparents pay insurance for children.

**Table 2:** Chi-squared test and OR results for common comorbidities of children with ADHD in relation to skipped-generation families.

Comorbidity	Yes (n = 381)	No (n = 78,782)	OR	95% CI	*p*-Value
Oppositional defiant disorder	31	6079	1.06	(0.73, 1.53)	0.830
Somatoform disorder	3	758	0.82	(0.26, 2.55)	0.930
Anxiety disorder	44	10,515	0.85	(0.62, 1.16)	0.340
Emotional disturbances during childhood and adolescence	25	3138	1.69	(1.13, 2.54)	0.015 *
Misery and unhappiness disorder	1	54	3.84	(0.53, 27.80)	0.650
Stereotypic movement disorder	0	52	---	---	1.000
Conduct disorder	11	1474	1.56	(0.85, 2.85)	0.200
Relationship problems	3	1231	0.50	(0.16, 1.60)	0.310
Learning disorder	13	2220	1.22	(0.70, 2.12)	0.590
Identity disorders during childhood and adolescence	0	5	---	---	1.000
Academic underachievement disorder	0	207	---	---	0.620
The adjustment reaction	18	1713	2.23	(1.39, 3.59)	<0.01 **
Autistic spectrum disorder	20	5641	0.72	(0.46, 1.13)	0.180
Bipolar disorder	7	509	2.88	(1.36, 6.11)	0.010 *
Shyness and introverted disorder in childhood	0	107	---	---	0.980
Acute reaction to stress	1	190	1.09	(0.15, 7.79)	1.000
Obsessive–compulsive disorder	2	370	1.12	(0.28, 4.47)	1.000
Personality disorder	2	45	9.23	(2.23, 38.20)	<0.01 **
Speech or language developmental disorder	19	5621	0.68	(0.43, 1.08)	0.130

*: *p* < 0.05, ** *p* < 0.05. ---: Odds ratio and 95% confidence interval were not estimable due to zero cases.

## Data Availability

The data used in this study are available from the National Health Insurance Research Database (NHIRD), maintained by the Health and Welfare Data Science Center (HWDC), Ministry of Health and Welfare, Taiwan. Due to the restrictions imposed by the “Personal Information Protection Act” of Taiwan, the data cannot be made publicly available. Requests for data access can be submitted as a formal proposal to the HWDC. More information is available at the HWDC official website (https://dep.mohw.gov.tw/dos/np-2497-113.html (accessed on 20 April 2024)) or by contacting stcarolwu@mohw.gov.tw.

## Supplementary Materials

The following supporting information can be downloaded at https://www.mdpi.com/article/10.3390/children12091123/s1, Table S1: Disease diagnostic coding and prescription. Table S2: Prevalence of comorbidities among children with ADHD across different age groups. Table S3: ORs of age-stratified various comorbidities in patients with ADHD compared with the control group. Table S4: T-test results of MACB for common ADHD comorbidities. Table S5: T-test results of PACB for common ADHD comorbidities. Table S6: ORs of the six statistically significant comorbidities stratified by MACB. Table S7: ORs of the six statistically significant comorbidities stratified by PACB. Table S8: STROBE Checklist. Figure S1: The annual incidence (2010–2013) of ADHD diagnosis in the overall pediatric and adolescent population in Taiwan and for age groups.

## Abbreviations

The following abbreviations are used in this manuscript:

ADHD	Attention deficit/hyperactivity disorder
ASD	Autism spectrum disorder
DMDD	Disruptive mood dysregulation disorder
DSM-IV	Diagnostic and Statistical Manual of Mental Disorders, Fourth Edition
ICD-9-CM	International Classification of Diseases, Ninth Revision, Clinical Modification
MACB	Maternal age at childbirth
NHIRD	National Health Insurance Research Database
OCD	Obsessive–compulsive disorder
ODD	Oppositional defiant disorder
ORs	Odds ratios
PACB	Paternal age at childbirth
