# Supplementary material for: Effect of Parental–Child Age Gaps and Skipped-Generation Families on Comorbidities Related to Attention Deficit Hyperactivity Disorder: A Population-Based Case–Control Study
Source: Children (Basel). 2025 Aug 26;12(9):1123. doi: 10.3390/children12091123 (PMC12468086; doi:10.3390/children12091123)
Supplement: Supplementary file 1 [file children-12-01123-s001.zip › children-3805274-supplementary.pdf]

Table S1. Disease diagnostic coding and prescription

**Comorbidities**	**ICD9 CM codes**
Learning disorder	315.00, 315.01, 315.02, 315.09, 315.1, 315.2, 784.60, 784.61, 784.69
Speech or language delay	3153,7843,78400, 78441 78449 7845
Autistic spectrum disorder	299
Obsessive-compulsive disorder	300.3
Anxiety disorder	300.00,300.01, 300.02, 300.21, 300.22, 300.23, 300.29, 30.03, 293.84, 313.0
Factitious disorder	300.16, 300.19
Dissociation	300.12, 300.13, 300.14, 300.15, 300.6
Somatoform disorder	300.81, 300.82, 300.11 307.80, 307.89, 300.7
Tourette syndrome	307.20, 307.21, 307.22, 307.23, 333.3
Stereotypic movement disorder	3073
Eating disorders	3071, 30751, 30750, 30752 30753 30759 7833
Psychogenic vomiting	307.54
Major depression	296.2, 296.3, 311, 300.4
Bipolar disorder	296.0, 296.1, 296.4, 296.5, 296.6, 296.7, 296.8, 296.9
Nocturnal enuresis and incontinence	307.6, 307.7, 787.6, 788.3, 788.43
Misery and unhappiness disorder	313.1
Shyness and Introverted disorder of childhood	313.21, 313.22
Selective mutism	313.23
Interpersonal difficulties	313.3
Oppositional defiant disorder	313,81
Identity disorder of childhood or adolescence	313.82
Academic underachievement disorder	313.83
Emotional disturbances of childhood or adolescent	313.89, 313.9
Sleep disorder	307.4, 347, 780.5, 327
Febrile seizure	780.31
Epilepsy and seizure disorders	345, 780.39
Sexual deviations and disorders	302
Alcohol dependence syndrome	303
Drug addiction	304
Substance abuse	305
Physiological malfunction arising from mental factors	306
Intellectual disability	317, 318, 319
Acute reaction to stress	308
Adjustment reaction	309

Specific nonpsychotic mental disorders due to brain damage	310
Conduct disorder	312
Psychic factors associated with diseases classified elsewhere	316
Schizophrenia	295, 297.1, 297.3, 298.8, 298.9, 293.18, 293.82, 293.89
Antisocial personality disorder	301.7
Other Personality disorders	301 (exclude 301.7)
Developmental coordination disorder	315.4
Skull fracture	800, 801, 802, 803, 804
Burn injury	940, 941, 942, 943, 944, 945, 946, 947, 948, 949
Traffic accident	E800-E848
Accidental falls	E881-E888
Accidents caused by fire and flames	E890-E899
Accidents due to natural and environmental factors	E900-E909
Accidents caused by submersion, suffocation and foreign bodies	E910-E915
Suicide and self-inflicted injury	E950-E959
Murder (Injury undetermined whether accidentally or purposely inflicted)	E980-E989

Table S2 Prevalence of comorbidities among children with ADHD across different age groups.

Comorbidities	ADHD					Controls				
	(0~6)	(7~12)	(13~18)	Overall	Proportion (%)	(0~6)	(7~12)	(13~18)	Overall	Proportion (%)
Anxiety disorder	699	6989	2871	10559	13.338	137	1172	1163	2472	0.625
Oppositional defiant disorder	232	3857	2021	6110	7.718	8	110	107	225	0.057
Intellectual disability	654	3484	1817	5955	7.522	388	2562	1134	4084	1.032
Autistic spectrum disorder	1071	3024	1566	5661	7.151	697	2253	790	3740	0.945
Speech or language disorder	3752	1705	183	5640	7.125	3480	3497	351	7328	1.851
Tourette syndrome	356	3475	1207	5038	6.364	674	5454	771	6899	1.743
Emotional disturbances of childhood or adolescent	498	1709	956	3163	3.996	133	660	381	1174	0.297
Epilepsy and seizure disorders	352	1427	554	2333	2.947	1021	4504	1477	7002	1.769
Learning disorder	130	1499	604	2233	2.821	125	599	144	868	0.219
Adjustment reaction	137	945	649	1731	2.187	38	426	573	1037	0.262
Conduct disorder	129	634	722	1485	1.876	61	315	192	568	0.144
Interpersonal difficulties	277	712	245	1234	1.559	96	311	66	473	0.12
Major depression	0	215	1002	1217	1.537	0	165	604	769	0.194
Sleep disorder	111	514	359	984	1.243	418	2276	1123	3817	0.964
Developmental coordination disorder	450	399	52	901	1.138	317	443	40	800	0.202
Somatoform disorder	9	458	294	761	0.961	1	76	72	149	0.038
Nocturnal enuresis and incontinence	46	460	60	566	0.715	248	2395	294	2937	0.742
Bipolar disorder	5	115	396	516	0.652	7	91	235	333	0.084

Obsessive-compulsive disorder	13	131	228	372	0.470	6	139	193	338	0.085
Physiological malfunction arising from mental factors	93	111	21	225	0.284	98	326	164	588	0.149
Schizophrenia	1	30	183	214	0.270	23	113	273	409	0.103
Academic underachievement disorder	5	109	93	207	0.261	1	70	36	107	0.027
Acute reaction to stress	43	114	34	191	0.241	16	64	55	135	0.034
Shyness and Introverted disorder of childhood	18	60	29	107	0.135	21	43	6	70	0.018
Selective mutism	7	63	12	82	0.104	15	66	19	100	0.025
Febrile seizure	36	26	7	69	0.087	847	361	9	1217	0.307
Head injury or skull fracture	8	38	12	58	0.073	1426	6128	3461	11015	2.783
Misery and unhappiness disorder	3	25	27	55	0.069	4	13	3	20	0.005
Stereotypic movement disorder	2	31	19	52	0.066	0	15	4	19	0.005
Eating disorders	7	12	31	50	0.063	54	129	44	227	0.057
Other personality disorder	5	13	29	47	0.059	1	17	30	48	0.012
Sexual deviations and disorders	0	4	19	23	0.029	0	10	25	35	0.009
Specific nonpsychotic mental disorders due to brain damage	2	11	10	23	0.029	37	293	238	568	0.144
Substance abuse	0	0	19	19	0.024	6	3	32	41	0.010
Burn injury	1	8	4	13	0.016	489	1535	601	2625	0.663
Drug addiction	0	0	6	6	0.008	0	5	10	15	0.004
Identity disorder of childhood or adolescence	0	3	2	5	0.006	0	0	2	2	0.001
Psychic factors associated with	0	3	2	5	0.006	2	19	8	29	0.007

diseases classified elsewhere										
Antisocial personality disorder	0	1	3	4	0.005	0	0	3	3	0.001
Skull fracture	2	2	0	4	0.005	146	770	986	1902	0.481
Accidental death	0	1	3	4	0.005	82	380	204	666	0.168
Alcohol dependence syndrome	0	0	3	3	0.004	0	0	12	12	0.003
Dissociation	0	0	2	2	0.003	0	3	6	9	0.002
Suicide and self-inflicted injury	0	0	2	2	0.003	0	2	13	15	0.004
Psychogenic vomiting	0	0	1	1	0.001	2	5	2	9	0.002
Accidents due to natural and environmental factors	0	0	1	1	0.001	33	225	112	370	0.093
Accidents caused by submersion, suffocation and foreign bodies	0	1	0	1	0.001	7	23	9	39	0.01
Injury undetermined whether accidentally or purposely inflicted	0	0	1	1	0.001	0	4	5	9	0.002
factitious disorder	0	0	0	0	0	0	3	3	6	0.002
Traffic accident	0	0	0	0	0	6	18	32	56	0.014
Accidental falls	0	0	0	0	0	18	64	24	106	0.027
Accidents caused by fire and flames	0	0	0	0	0	1	1	2	4	0.001
Murder	0	0	0	0	0	2	15	18	35	0.009

Table S3 ORs of age-stratified various comorbidities in patients with ADHD compared with the control group

Age groups	0~6		7~12		13~18		Total	
**Comorbidities**	**OR**	**95%CI**	**OR**	**95%CI**	**OR**	**95%CI**	**OR**	**95%CI**
Anxiety disorder	26.57	(22.11,31.93)	35.04	(32.91,37.31)	14.69	(13.69,15.76)	24.49	(23.42,25.61)
Oppositional defiant disorder	147.01	(72.64,297.53)	191.45	(158.35,231.47)	107.24	(88.22,130.36)	147.05	(128.70,168.02)
intellectual disability	8.73	(7.69,9.91)	7.28	(6.91,7.67)	8.86	(8.21,9.57)	7.80	(7.49,8.13)
Autistic spectrum disorder	8.14	(7.39,8.97)	7.12	(6.73,7.52)	10.83	(9.92,11.82)	8.07	(7.74,8.42)
Speech or language disorder	6.65	(6.33,6.99)	2.49	(2.35,2.64)	2.62	(2.19,3.14)	4.07	(3.92,4.21)
Tourette syndrome	2.68	(2.35,3.05)	3.37	(3.22,3.52)	8.36	(7.62,9.16)	3.83	(3.69,3.98)
Emotional disturbances of childhood or adolescent	19.26	(15.89,23.34)	13.41	(12.25,14.68)	13.24	(11.75,14.93)	13.99	(13.08,14.97)
Epilepsy and seizure disorders	1.74	(1.54,1.97)	1.60	(1.51,1.70)	1.91	(1.73,2.10)	1.69	(1.61,1.77)
Learning disorder	5.23	(4.09,6.69)	12.90	(11.73,14.19)	21.72	(18.09,26.07)	13.21	(12.21,14.29)
Adjustment reaction	18.17	(12.68,26.04)	11.31	(10.08,12.68)	5.85	(5.22,6.55)	8.51	(7.88,9.19)
Conduct disorder	10.65	(7.85,14.45)	10.19	(8.90,11.67)	19.60	(16.70,23.01)	13.30	(12.07,14.66)
Interpersonal difficulties	14.65	(11.61,18.49)	11.61	(10.16,13.27)	18.82	(14.33,24.71)	13.24	(11.90,14.72)
Major depression	NA	NA	6.54	(5.34,8.01)	8.76	(7.91,9.70)	8.02	(7.33,8.78)
Sleep disorder	1.33	(1.08,1.64)	1.13	(1.03,1.24)	1.61	(1.43,1.82)	1.29	(1.20,1.39)
Developmental coordination disorder	7.26	(6.28,8.39)	4.53	(3.96,5.19)	6.52	(4.31,9.85)	5.68	(5.17,6.25)
Somatoform disorder	45.02	(5.70,355.38)	30.43	(23.86,38.80)	20.76	(16.04,26.88)	25.78	(21.62,30.73)
Nocturnal enuresis and incontinence	0.93	(0.68,1.27)	0.96	(0.87,1.06)	1.02	(0.77,1.35)	0.96	(0.88,1.05)
Bipolar disorder	3.57	(1.13,11.25)	6.33	(4.81,8.34)	8.61	(7.32,10.12)	7.79	(6.79,8.94)
Obsessive-compulsive disorder	10.84	(4.12,28.52)	4.72	(3.72,6.00)	5.97	(4.93,7.24)	5.52	(4.77,6.40)
Physiological malfunction arising	4.77	(3.59,6.34)	1.70	(1.37,2.11)	0.64	(0.41,1.01)	1.92	(1.64,2.23)

from mental factors								
Schizophrenia	0.22	(0.03,1.63)	1.33	(0.89,1.99)	3.38	(2.80,4.08)	2.62	(2.22,3.09)
Academic underachievement disorder	25.01	(2.92,214.09)	7.80	(5.78,10.54)	12.98	(8.83,19.08)	9.70	(7.68,12.25)
Acute reaction to stress	13.47	(7.59,23.92)	8.93	(6.57,12.13)	3.10	(2.02,4.75)	7.09	(5.69,8.84)
Shyness and Introverted disorder of childhood	4.29	(2.29,8.05)	6.98	(4.72,10.33)	24.21	(10.05,58.31)	7.65	(5.66,10.34)
Selective mutism	2.33	(0.95,5.72)	4.78	(3.38,6.75)	3.16	(1.53,6.51)	4.10	(3.06,5.50)
Febrile seizure	0.21	(0.15,0.29)	0.36	(0.24,0.54)	3.89	(1.45,10.45)	0.28	(0.22,0.36)
Head injury or skull fracture	0.03	(0.01,0.06)	0.03	(0.02,0.04)	0.02	(0.01,0.03)	0.03	(0.02,0.03)
Misery and unhappiness disorder	3.75	(0.84,16.76)	9.62	(4.92,18.81)	45.07	(13.67,148.59)	13.76	(8.25,22.95)
Stereotypic movement disorder	0.5	(0.13,2.00)	10.34	(5.58,19.15)	23.78	(8.09,69.90)	13.69	(8.10,23.16)
Eating disorders	0.65	(0.30,1.43)	0.46	(0.26,0.84)	3.53	(2.23,5.59)	1.10	(0.81,1.50)
Other personality disorder	25.01	(2.92,214.09)	3.82	(1.86,7.87)	4.84	(2.90,8.07)	4.90	(3.28,7.32)
Sexual deviations and disorders	NA	NA	2.00	(0.63,6.38)	3.80	(2.09,6.91)	3.29	(1.94,5.56)
Specific nonpsychotic mental disorders due to brain damage	0.27	(0.07,1.12)	0.19	(0.10,0.34)	0.21	(0.11,0.39)	0.20	(0.13,0.31)
Substance abuse	NA	NA	NA	NA	2.97	(1.68,5.24)	2.32	(1.35,3.99)
Burn injury	0.01	(0.00,0.07)	0.03	(0.01,0.05)	0.03	(0.01,0.09)	0.02	(0.01,0.04)
Drug addiction	NA	NA	NA	NA	3.00	(1.09,8.26)	2.00	(0.78,5.16)
Identity disorder of childhood or adolescence	NA	NA	0.50	(0.16,1.55)	5.00	(0.70,35.50)	12.50	(2.43,64.44)
Psychic factors associated with diseases classified elsewhere	NA	NA	0.79	(0.23,2.67)	1.25	(0.27,5.89)	0.86	(0.33,2.23)
Antisocial personality disorder	NA	NA	0.50	(0.07,3.55)	5.00	(1.01,24.78)	6.67	(1.49,29.79)
Skull fracture	0.069	(0.02,0.28)	0.01	(0.00,0.05)	NA	NA	0.01	(0.00,0.03)

Accidental death	NA	NA	0.01	(0.00,0.09)	0.07	(0.02,0.23)	0.03	(0.01,0.08)
Alcohol dependence syndrome	NA	NA	NA	NA	1.25	(0.35,4.43)	1.25	(0.35,4.43)
Dissociation	NA	NA	NA	NA	1.67	(0.34,8.26)	1.11	(0.24,5.14)
Suicide and self-inflicted injury	NA	NA	NA	NA	0.77	(0.17,3.41)	0.67	(0.15,2.92)
Psychogenic vomiting	NA	NA	NA	NA	2.50	(0.23,27.57)	0.56	(0.07,4.39)
Accidents due to natural and environmental factors	NA	NA	NA	NA	0.04	(0.01,0.32)	0.01	(0.00,0.10)
Accidents caused by submersion, suffocation and foreign bodies	NA	NA	0.22	(0.03,1.61)	NA	NA	0.13	(0.02,0.93)
Injury undetermined whether accidentally or purposely inflicted	NA	NA	NA	NA	1.00	(0.12,8.56)	0.56	(0.07,4.39)
factitious disorder	NA	NA	NA	NA	NA	NA	NA	NA
Traffic accident	NA	NA	NA	NA	NA	NA	NA	NA
Accidental falls	NA	NA	NA	NA	NA	NA	NA	NA
Accidents caused by fire and flames	NA	NA	NA	NA	NA	NA	NA	NA
Murder	NA	NA	NA	NA	NA	NA	NA	NA

Table S4 T-test results of MACB for common ADHD comorbidities (**p<0.01, *p<0.05)

	Mean age	SD	Number	T value	p value
Oppositional defiant disorder (y)	29.67	4.87	2951	1.28	0.2
Oppositional defiant disorder (n)	29.55	4.74	33653		
Somatoform disorder (y)	29.3	4.23	339	-1.14	0.26
Somatoform disorder (n)	29.56	4.75	36165		
Anxiety disorder (y)	29.02	4.71	4930	-8.49	**<0.01
Anxiety disorder (n)	29.64	4.75	31574		
Emotional disturbances of childhood or adolescent (y)	29.44	4.84	1410	-0.95	0.34
Emotional disturbances of childhood or adolescent (n)	29.56	4.75	35094		
Misery and unhappiness disorder (y)	29.3	4.05	20	-1.19	0.24
Misery and unhappiness disorder (n)	29.56	4.75	36484		
Stereotypic movement disorder (y)	27.92	4.11	25	1.73	0.08
Stereotypic movement disorder (n)	29.56	4.75	36479		
Conduct disorder (y)	29.15	5.34	597	-1.9	0.06
Conduct disorder (n)	29.56	4.74	35907		
Relationship problems (y)	29.53	4.5	582	-0.16	0.88
Relationship problems (n)	29.56	4.75	35922		
Learning disorder (y)	29.4	4.86	1002	-1.06	0.29
Learning disorder (n)	29.56	4.75	35502		
Sexual deviations and disorders	35	1.41	2	1.62	0.11
Sexual deviations and disorders	29.56	4.75	36502		
Academic underachievement disorder (y)	28.77	4.21	83	-1.51	0.13
Academic underachievement disorder (n)	29.56	4.75	36421		
Adjustment reaction (y)	28.72	4.76	741	-4.87	**<0.01

Adjustment reaction (n)	29.58	4.75	35763		
Autistic spectrum disorder (y)	30.48	4.75	2531	10.13	**<0.01
Autistic spectrum disorder (n)	29.49	4.74	33973		
Bipolar disorder (y)	28.69	4.78	189	-2.51	*0.01
Bipolar disorder (n)	29.56	4.75	36315		
Shyness and Introverted disorder of childhood (y)	29.48	4.5	44	-0.11	0.91
Shyness and Introverted disorder of childhood (n)	29.56	4.75	36460		
Acute reaction to stress (y)	30.23	5.22	84	1.29	0.2
Acute reaction to stress (n)	29.56	4.75	36420		
Antisocial personality disorder (y)	26	NA	1	NA	NA
Antisocial personality disorder (n)	29.56	4.75	36503		
Obsessive-compulsive disorder (y)	30.24	4.99	178	1.91	0.06
Obsessive-compulsive disorder (n)	29.55	4.75	36326		
Other personality disorder (y)	31.16	7.4	19	0.94	0.36
Other personality disorder (n)	29.56	4.75	36485		
Speech or language disorder (y)	30.11	4.94	2493	5.75	<0.01
Speech or language disorder (n)	29.52	4.73	34011		

Table S5 T-test results of PACB for common ADHD comorbidities (**p<0.01, *p<0.05)

	Mean age	SD	Number	T value	p value
Oppositional defiant disorder (y)	32.39	5.65	2233	-0.54	0.59
Oppositional defiant disorder (n)	32.45	5.61	29517		
Somatoform disorder (y)	31.92	5.17	282	-1.6	0.11
Somatoform disorder (n)	32.45	5.62	31468		
Anxiety disorder (y)	32.14	5.75	4087	-3.71	**<0.01
Anxiety disorder (n)	32.5	5.6	27663		
Emotional disturbances of childhood or adolescent (y)	32.34	5.8	1168	-0.69	0.49
Emotional disturbances of childhood or adolescent (n)	32.45	5.6	30582		
Misery and unhappiness disorder (y)	31.89	5.66	18	-0.42	0.67
Misery and unhappiness disorder (n)	32.45	5.61	31732		
Stereotypic movement disorder (y)	30.1	5.74	21	-1.88	0.07
Stereotypic movement disorder (n)	32.45	5.61	31729		
Conduct disorder (y)	32.14	6.03	543	-1.2	0.23
Conduct disorder (n)	32.46	5.6	31207		
Relationship problems (y)	32.44	5.76	499	-0.04	0.97
Relationship problems (n)	32.45	5.61	31251		
Learning disorder (y)	32.26	5.95	853	-0.94	0.35
Learning disorder (n)	32.46	5.6	30897		
Sexual deviations and disorders	26	4.24	2	-1.63	0.1
Sexual deviations and disorders	34.45	5.61	31748		
Academic underachievement disorder (y)	31.55	4.92	88	-1.51	0.13
Academic underachievement disorder (n)	32.45	5.61	31662		
Adjustment reaction (y)	31.76	5.52	665	-3.19	**<0.01
Adjustment reaction (n)	32.46	5.61	31085		
Autistic spectrum disorder (y)	33.54	5.53	2504	10.18	**<0.01

Autistic spectrum disorder (n)	32.36	5.61	29246		
Bipolar disorder (y)	32.39	6.08	176	-0.15	0.88
Bipolar disorder (n)	32.45	5.61	31574		
Shyness and Introverted disorder of childhood (y)	32.35	5	54	-0.13	0.9
Shyness and Introverted disorder of childhood (n)	32.45	5.61	31696		
Acute reaction to stress (y)	33.13	5.73	72	1.02	0.31
Acute reaction to stress (n)	32.45	5.61	31678		
Antisocial personality disorder (y)	40	NA	1	NA	NA
Antisocial personality disorder (n)	32.45	5.61	31749		
Obsessive-compulsive disorder (y)	33.41	5.26	149	2.09	*0.04
Obsessive-compulsive disorder (n)	32.45	5.61	31601		
Other personality disorder (y)	33.8	7.71	10	0.76	0.45
Other personality disorder (n)	32.45	5.61	31740		
Speech or language disorder (y)	33.27	5.8	2447	7.29	**<0.01
Speech or language disorder (n)	32.38	5.59	29303		

Table S6. ORs of the six statistically significant comorbidities stratified by MACB

Maternal age	Anxiety	No anxiety	OR	95%CI
≦20	130	770	1.05	(0.87,1.28)
21-25	1016	5159	1.23	(1.13,1.33)
26-30	2009	12529	1.00	ref
31-35	1339	9718	0.86	(0.80,0.93)
>35	436	3398	0.80	(0.72,0.89)

Maternal age	Adjust reaction	No adjust reaction	OR	95%CI
≦20	27	873	1.41	(0.95,2.11)
21-25	157	6018	1.19	(0.98,1.45)
26-30	311	14227	1.00	ref
31-35	182	10875	0.77	(0.64,0.92)
>35	64	3770	0.78	(0.59,1.02)

Maternal age	OCD	No OCD	OR	95%CI
≦20	4	896	0.90	(0.33,2.46)
21-25	22	6153	0.72	(0.45,1.16)
26-30	72	14466	1.00	ref
31-35	57	11000	1.04	(0.73,1.48)
>35	23	3811	1.21	(0.76,1.94)

Maternal age	Autism	No autism	OR	95%CI
≦20	45	855	0.76	(0.56,1.04)
21-25	322	5853	0.80	(0.70,0.91)
26-30	938	13600	1.00	ref
31-35	873	10184	1.24	(1.13,1.37)
>35	353	3481	1.47	(1.29,1.67)

Maternal age	Speech or language delay	No speech or language delay	OR	95%CI
≦20	57	843	0.97	(0.74,1.28)
21-25	366	5809	0.91	(0.80,1.03)
26-30	944	13594	1.00	ref
31-35	802	10255	1.13	(1.02,1.24)
>35	324	3510	1.33	(1.17,1.52)

Maternal age	Bipolar disorder	No Bipolar disorder	OR	95%CI
≦20	10	890	2.01	(1.04,3.88)
21-25	34	6141	0.99	(0.66,1.48)
26-30	81	14457	1.00	ref
31-35	50	11007	0.81	(0.57,1.15)
>35	14	3820	0.65	(0.37,1.15)

Table S7. ORs of the six statistically significant comorbidities stratified by PACB

Paternal age	Anxiety	No anxiety	OR	95%CI
≦20	45	225	1.39	(1.01,1.93)
21-25	356	2160	1.15	(1.01,1.30)
26-30	1261	8066	1.09	(1.00,1.18)
31-35	1415	9851	1.00	ref
>35	1010	7361	0.96	(0.88,1.04)

Paternal age	Adjust reaction	No adjust reaction	OR	95%CI
≦20	6	264	1.12	(0.49,2.54)
21-25	71	2445	1.43	(1.09,1.88)
26-30	212	9115	1.15	(0.95,1.39)
31-35	224	11042	1.00	ref
>35	152	8219	0.91	(0.74,1.12)

Paternal age	OCD	No OCD	OR	95%CI
≦20	2	268	1.37	(0.33,5.64)
21-25	4	2512	0.29	(0.11,0.81)
26-30	34	9293	0.67	(0.44,1.02)
31-35	61	11205	1.00	ref
>35	48	8323	1.06	(0.73,1.55)

Paternal age	Autism	No autism	OR	95%CI
≦20	11	259	0.45	(0.25,0.83)
21-25	119	2397	0.53	(0.44,0.64)
26-30	598	8729	0.73	(0.66,0.81)
31-35	966	10300	1.00	ref
>35	810	7561	1.14	(1.04,1.26)

Paternal age	Speech or language delay	No speech or language delay	OR	95%CI
≦20	17	253	0.78	(0.47,1.28)
21-25	138	2378	0.67	(0.56,0.81)
26-30	641	8686	0.86	(0.77,0.95)
31-35	895	10371	1.00	ref
>35	756	7615	1.15	(1.04,1.27)

Paternal age	Bipolar disorder	No Bipolar disorder	OR	95%CI
≦20	6	264	3.58	(1.54,8.32)
21-25	12	2504	0.76	(0.41,1.40)
26-30	45	9282	0.76	(0.53,1.11)
31-35	71	11195	1.00	ref
>35	42	8329	0.80	(0.54,1.17)

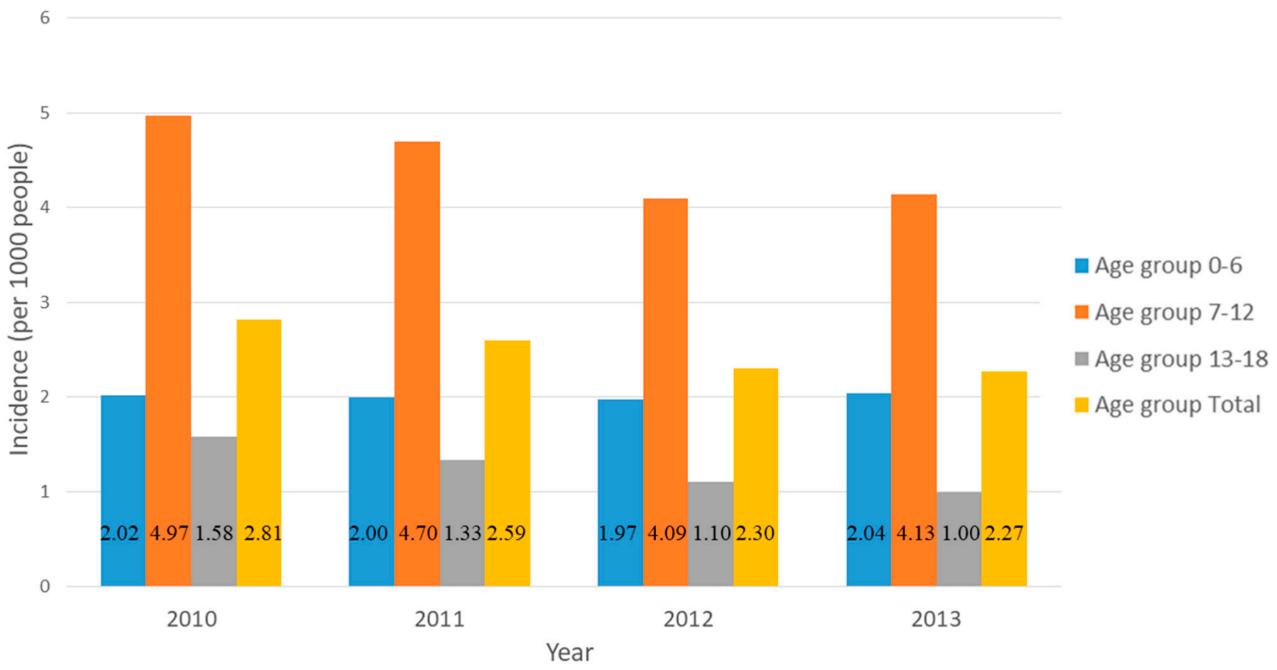

Supp Figure S1. The annual incidence (2010–2013) of ADHD diagnosis in the overall pediatric and adolescent population in Taiwan and their age groups.

Table S8 STROBE Checklist

Section / Item	Recommendation	Addressed in Manuscript
**Title and Abstract**	1. Indicate study design in title/abstract; provide informative summary.	**Yes –** Title specifies "population-based case–control study"; abstract includes objectives, methods, results, and conclusions.
**Introduction**	2. Explain scientific background and rationale.	**Yes –** ADHD comorbidities, parental age, and skip-generation families described.
	3. State objectives and hypotheses.	**Yes –** Hypotheses about parental age/skip-generation caregiving stated.
**Methods**	4. Present key elements of study design early.	**Yes –** Study design is clear, and is emphasized in the introduction.
	5. Describe setting, locations, relevant dates.	**Yes –** Taiwan, NHIRD, 2009–2013.
	6. Describe eligibility criteria, sources/methods of case/control selection.	**Yes –** Cases: ADHD children <18; Controls: matched 1:5 by age/sex.
	7. Clearly define variables (outcomes, exposures, confounders).	**Yes –** Outcomes: 20 comorbidities; Exposures: parental age, skip-generation caregiving.
	8. Data sources and measurement methods.	**Yes –** NHIRD database; diagnoses validated by neurologists and psychiatrist.
	9. Efforts to address potential bias.	**Partial –** Diagnostic misclassification acknowledged, SES/urban–rural confounding discussed; no formal adjustment performed.
	10. Study size and rationale.	**Yes**- Very large NHIRD cohort.
	11. Explain handling of quantitative variables.	**Yes –** Parental age categorized; comorbidities coded via ICD-9-CM.
	12. Statistical methods.	**Yes –** Logistic regression, chi-square, t-tests; stratified analyses. Multiple testing correction not applied.
**Results**	13. Report numbers of individuals at each stage.	**Yes –** Cases (79,163) and controls (395,815) clearly reported.
	14. Give characteristics of study participants.	**Yes –** Demographics in Table 1.
	15. Report outcome data for each exposure.	**Yes –** Figures 1–5, Tables S2–S7.

Section / Item	Recommendation	Addressed in Manuscript
	16. Main results (unadjusted/adjusted estimates, CIs).	**Yes –** ORs with 95% CIs provided.
	17. Other analyses (subgroups, sensitivity analyses).	**Partial –** Age-stratified analyses performed; no SES or sensitivity analyses.
**Discussion**	18. Summarize key results with reference to objectives.	**Yes –** Extremes of parental age and skip-generation caregiving linked to specific comorbidities.
	19. Discuss limitations.	**Yes –** Diagnostic misclassification, wide CIs, no multiple testing correction, unmeasured confounding.
	20. Interpretation of results.	**Yes –** Compared to prior studies, biological/psychosocial mechanisms discussed.
	21. Generalisability of findings.	**Yes –** Applicability to Taiwan and regions with similar family structures discussed.
**Other Information**	22. Funding, ethical approval, conflicts of interest.	**Yes –** Funding source (TTMHH-R114-0095), IRB approval, consent waiver, no conflicts declared.
